# Exploring the potential role of sonic hedgehog cell signalling pathway in antidepressant effects of nicotine in chronic unpredictable mild stress rat model

**DOI:** 10.1016/j.heliyon.2019.e01600

**Published:** 2019-05-10

**Authors:** Mohd Tayyab, Mehdi H. Shahi, Shirin Farheen, Mubeena Mariyath P.M., Nabeela Khanam, M. Mobarak Hossain

**Affiliations:** a Interdisciplinary Brain Research Centre, J.N. Medical College, Faculty of Medicine, Aligarh Muslim University, Aligarh, Uttar Pradesh 202002, India; b Department of Physiology, J.N. Medical College, Faculty of Medicine, Aligarh Muslim University, Aligarh, Uttar Pradesh 202002, India

**Keywords:** Neuroscience

## Abstract

Nicotine is the most common and highly addictive drug of abuse, associated with several life-threatening diseases and high mortality. Nicotine abuse is the concerted effort to feel reward and fight depression in depressed individuals. The underlying mechanism of nicotine is to activate the brain reward system in the central nervous system and provide an antidepressant effect. Antidepressants provide their therapeutic effect by stimulating hippocampal neurogenesis, which can be correlated with brain derived neurotrophic factor (BDNF) expression in the hippocampus. BDNF interacts with Wnt/β-catenin and sonic hedgehog (Shh) signalling cascade to stimulate hippocampal neurogenesis. Shh is the marker of hippocampal neurogenesis and also involved in the neuropathology of depression. But knowledge in this area to identify the potential therapeutic target is limited. In our study, we explored the role of BDNF, Wnt/β-catenin and Shh signalling in depression and the involvement of these signalling pathways in providing an antidepressant effect by nicotine. Our investigations showed that chronic unpredictable mild stress induced depression results declined expression of BDNF, Wnt/β-catenin, Shh and its downstream transcription factors GLI1/2/3 and NKX2.2 in the hippocampus of male Wistar rat. Moreover, we also observed that nicotine administration increased the expression of these signalling molecules in providing the antidepressant effects.

## 1 Introduction

Nicotine (NIC) is addictive and the most common drug of abuse either through smoking or by consumption of tobacco containing products. It is highly associated with various diseases and mortality [1]. According to the World Health Organization report on the Global Tobacco Epidemic (2017), smoking related mortality has risen to more than 7 million per year, which is higher than HIV/AIDS, malaria and tuberculosis combined [2]. Various epidemiological studies showed that NIC addiction is almost twice in clinically depressed individuals and they are more resistance to quit smoking as compared to non-depressed population [3]. Depression is one of the most prevalent mental health concern, affecting more than 322 million people which makes it significant disease burden and may become the leading cause of long-term disability by 2030 [4].

It has been reported that exposure to stressors leads to urge for smoking cigarettes [5]. The neurophysiology of NIC addiction is same as cocaine, opiates and alcohol [6]. The reason of desire to smoke is because NIC provides rewarding and motivational effects through acting on ventral tegmental area (VTA) and nucleus accumbens, as these areas are crucial for motivation and reward processing in the central nervous system (CNS) [6, 7]. A recent study suggested that VTA is also involved in depression and antidepressant medication can stimulate reward processing system and modulate emotion related behavior [8]. Due to its psychoactive properties [9], NIC stimulates CNS, which provides antidepressant-like effects [10, 11, 12], and therefore, enhance cognitive ability including learning and memory [10, 13].

In addition, studies suggested that NIC increases levels of neuronal growth factors in depression [14]. Brain derived neurotrophic factor (BDNF) is the most abundant neurotrophin and principal neuronal growth factor affected by NIC [14]. BDNF is involved in synaptic plasticity and hippocampal neurogenesis in the brain [15]. Neurotrophin theory of depression suggests that decreased BDNF level can cause reduced hippocampal neural proliferation, which results in the pathophysiology of depressive disorder including cognitive deficits [16]. Moreover, some antidepressants reported to show their therapeutic effect by enhancing hippocampal BDNF expression [17].

Another important signalling pathway involved in depression is Wnt/β-catenin signalling [18]. In which GSK-3β phosphorylates β-catenin and lead to its proteosomal degradation and restricts further gene transcription. In stressful condition especially in depression β-catenin expression downregulates [19, 20]. Moreover, chronic electroconvulsive therapy upregulates β-catenin expression in the hippocampus and stimulate hippocampal neurogenesis even in severe depression like drug resistant depression [19]. In our study we are the first to report that NIC also upregulate hippocampal β-catenin expression in depression.

Moreover, there are some studies in recent years which suggested that sonic hedgehog (Shh) siganlling also involve in the depression [21, 22, 23, 24] and BDNF is regulated by Wnt/β-catenin as well as Shh signalling [25, 26]. A recent research suggested a crosstalk between BDNF, Wnt/β-catenin and Shh signaling in chronic unpredictable mild stress (CUMS) induced prenatal stress and reported that the BDNF, β-catenin and Shh downregulates in depression and also decreases hippocampal neurogenesis [24]. Shh plays crucial role in adult hippocampal neurogenesis [27] and also a critical target in various neurological diseases [28]. Shh acts via its receptors patch1 and smoothened which further activates its primary downstream siganlling component zinc finger transcription factor GLI1/GLI2 (GLI1/2) as a transcriptional activator and GLI3 acts as transcriptional repressor in a gradient manner [28]. Further, Shh-GLI1/2 cell signalling regulate the transcription of homedomain transcription factors NKX2.2 and PAX6 [28]. The effect of NIC on Shh and Wnt/β-catenin expression in the hippocampus during stress is not studied so far, although, smoking rate during stress or depression is high. More investigation is required to explore molecular mechanism of NIC in neuropathology of depression to identify novel therapeutic targets to treat depression.

The *ex-vivo* study was aimed to investigate the potential effects of NIC on neurodevelopmental pathways such as BDNF, Wnt/β-catenin and Shh signalling pathways in CUMS induced depression and its associated cognitive deficits in rat brain.

## 2 Materials & methods

### 2.1 Reagents

Nicotine (Purity ≥99%, Cat no. N3876-100ML) was purchased from Sigma-Aldrich, Inc., (MO, USA). TRI reagent (Cat no. T9424-100ML) and Ethidium Bromide (Cat no. E8751-1G) was also purchased from Sigma-Aldrich, Inc., (MO, USA). M-MLV Reverse Transcriptase (Cat no. AM2043) and Random Hexamers (Cat no. SO142) used for cDNA synthesis was purchased from Invitrogen Bioservices India Pvt. Ltd., dNTP Mix (10mM each, Cat no. R0192), RiboLock RNase Inhibitor (Cat no. EO0381) and PCR Master Mix (Cat no. K0171) and Sybergreen Master Mix (Cat no. 4367659) was purchased from Thermo Scientific (India). Primers were synthesized from eurofin (India).

### 2.2 Animals

Adult male Wistar rats were used in the experiments, weighing 170–220g (3 months old). Rats were obtained from Central Animal House Facility, Jawaharlal Nehru Medical College (JNMC), Faculty of Medicine, Aligarh Muslim University (AMU) and were acclimated to the Interdisciplinary Brain Research Centre, Faculty of Medicine, AMU, animal house facility for 1 week prior to the experiments under controlled conditions (12 h light/dark cycle, lights on/off time 6:00 AM to 6:00 PM, room temperature 21 ± 2 °C and 3 rats per cage) with the free access to the food and water. All the behavioral experiments were carried out between 10 am to 5 pm. All the experiments in this study were carried out in accordance with the regulation of Institutional Animal Ethics Committee (IAEC) and were approved by IAEC (Registration No. 401/RO/c/2001/CPCSEA), Central Animal House, JNMC, Faculty of Medicine, AMU, Aligarh, U.P. India. All the experiments were done under the guidelines of CPCSEA, India.

Rats were randomly assigned to four groups; each group consists of 6 rats. (1) Control group (CON) received physiological saline for 4 weeks; (2) NIC group (Positive control) received NIC at a dose of 0.3 mg/kg/day for 4 weeks; (3) CUMS group was given physiological saline for 4 weeks; and (4) CUMS + NIC group were administered with NIC 0.3 mg/kg/day for 4 weeks.

### 2.3 Drug treatment

The preferred dose of NIC was 0.3 mg/kg as per the previous studies [29, 30]. NIC was dissolved in saline (0.9% NaCl) and was administered subcutaneously (s.c.) at pH 7. Fresh NIC solution was prepared every day for the administration.

### 2.4 Chronic unpredictable mild stress procedure

**Fig. 1:**
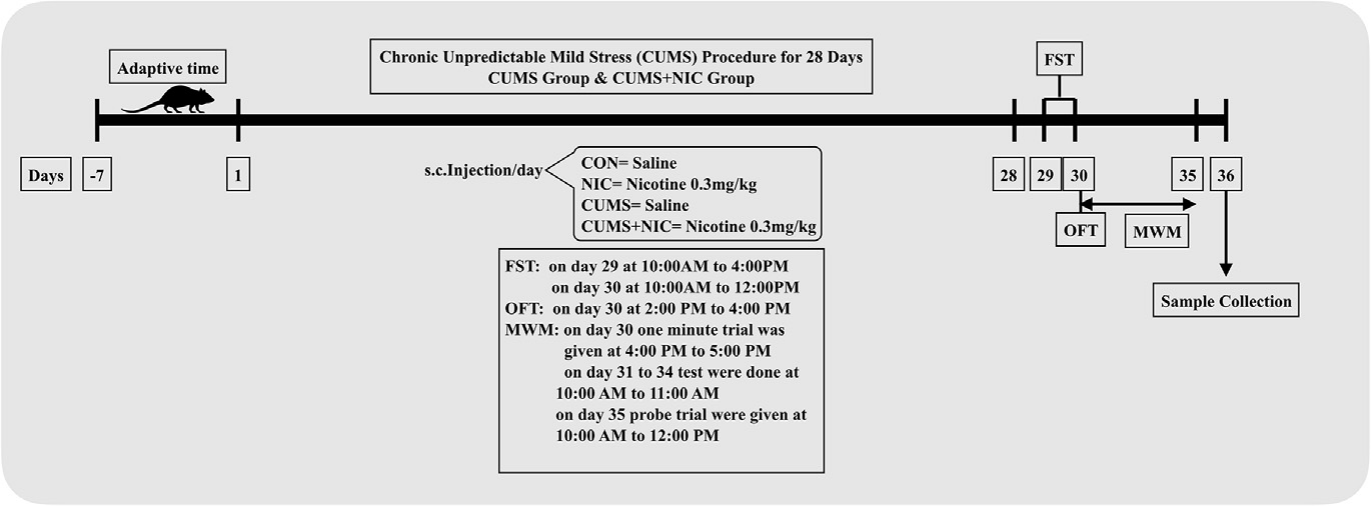
Schematic representation of experimental design. After 28 days of CUMS procedure FST, OFT and MWM were carried and thereafter, rat brain hippocampus samples were collected by animals sacrifice.

The CUMS protocol was performed as described previously [31] with slight modification in the procedure. Rats were subjected to the several mild stressors for 4 weeks (Fig. 1), a different mild stressor every day makes the procedure unpredictable, scheduled in such a fashion that the same will not repeat in the same week. There were a total of seven stressors: (i) food deprivation for 24 h; (ii) soiled bedding (∼150 ml water per cage) for 22 h; (iii) cage tilting (∼45°) for 22 h; (iv) crowded housing (12 animals per cage) for 12 h; (v) restraint stress for 2 h; (vi) water deprivation for 24 h; (vii) forced swimming for 10 min. Non stressed rats were left undisturbed in a separate room in their home cages.

### 2.5 Behavioral tests

To observe the behavioral effects of NIC on CUMS rats were subjected to different behavioral tests including open field test (OFT) for exploratory behavior and force swim test (FST) for behavioral despair. Also, Morris water maze (MWM) was conducted to assess the effect of NIC and CUMS on memory and learning. All the behavioral tests were analysed with ANY-maze, V4.3 (Stoelting, IL, USA).

### 2.6 Forced swim test

The FST was first described by Porsolt [32]. Briefly, each rat was placed in a glass cylinder of height 45 cm and diameter of 15 cm, filled with water up to 35 cm high of 25 ± 2 °C. The rat could not climb over the ridges or touch its tail at the bottom of the cylinder and was forced to swim. The test was the two-day procedure, at first day 15 min trial was given (no data collected) and on the second day the final test was conducted for the 5 min and the behavior was recorded by the camera placed overhead. The rat was considered immobile when it stopped struggling, which is a sign of behavioral despair. At last all rats were wiped with a sterile cloth and kept in their respective cages. The water was changed after each test.

### 2.7 Open field test

The OFT was performed as previously described [33] with slight modification [34]. The OFT arena consisted of 60 × 60 cm black wooden apparatus. The arena is surrounded by the 30 cm high walls and equally divided into 15 × 15 cm squares on the floor by white colored lines. It was the two-day procedure, on the first day 5 min trial was given and next day the final test was conducted. The test was started by placing the rat at the center of the arena and allowed it to explore freely for 5 min and the behavior was recorded by the overhead camera attached to ANY-Maze system V4.3 (Steolting, IL, USA). After each trial, the apparatus was cleaned with 70 percent ethanol.

### 2.8 Morris water maze

MWM was conducted to assess memory and learning as described previously [35]. The MWM tank was 132 cm in diameter and 60 cm in height, filled with water (25 ± 2 °C) to the depth of 45 cm. The dye was added to make the water opaque. The tank was divided into four equal quadrants by two virtual perpendicular lines crossing at the center. The four quadrant was named as southwest (SW), northwest (NW), northeast (NE) and southeast (SE). A platform of 10 cm diameter was placed in the middle of the target quadrant (SW) of the tank. The platform was submerged 2.5 cm below the surface of water for the rat to climb on it, to escape from the water. The MWM test completed in six days, on the first day the rats were trained to remember the visible platform and from the second day the platform was hidden to record escape latency and give three trials each day by placing the rat in different quadrants (NW, NE and SE) up to five days. The duration of each trial was 60 s only. If the rat was not able to find the platform in 60 s, it was placed on the platform by the experimenter to remind it the position of platform in the tank for 30 s. Thereafter, on the sixth day the probe trial was carried out. In the probe trial the platform was removed and allowed the rat to search the platform for 60 s, during which the time spent in the target quadrant was recorded by the ANY-Maze system, V4.3 (Steolting, IL, USA). After the end of each trial, the rat was soaked with the towel and placed them in their home cages.

### 2.9 Tissue sampling

After completion of behavioral experiments, rats were anaesthetized by chloral hydrate [36, 37] 400 mg/kg body weight and thereafter sacrificed. The brain of rats was dissected and placed on phosphate buffer saline immediately and hippocampus was isolated from the whole brain. RNA*later* RNA stabilization reagent (Qiagen) was added in the isolated tissue samples and stored at -20 °C for molecular analysis.

### 2.10 Reverse transcriptase PCR and real-time PCR

The qRTPCR were performed as described by *Schmittgen et al.* [38]. Total mRNA isolation was done by TRI reagent by following the manufacturer's instructions. The cDNA was synthesized by M-MLV Reverse Transcriptase. The reverse transcriptase PCR (RT-PCR) and real-time PCR (qRT-PCR) was done from the cDNA. βactin was used as housekeeping gene and the target genes were; BDNF, Shh, NKX2.2, PAX6, GLI1/2/3 and β-catenin. Gene expression was normalized using β actin as reference gene. The expression of mRNA was first observed by RT-PCR followed by agarose gel electrophoresis and the analysis was carried out by using the FluorChem E system CA, USA. Thereafter, qRT-PCR was conducted on LightCycler 480 Instrument (Roche).

### 2.11 Statistical analysis

All the behavioral test data were presented as mean ± SEM, analyzed by two-way ANOVA multiple comparisons and post-hoc Tukey test was used. For escape latency repeated measure two-way ANOVA was used. For qRT-PCR one-way ANOVA multiple comparision and post-hoc Tuckey test was used. Graphpad Prism 7 (for Mac OSX) was used for the analysis. A value of P < 0.05 was regarded as significant.

## 3 Results

### 3.1 Nicotine administration reverses the depressive-like behaviors induced by CUMS

**Fig. 2:**
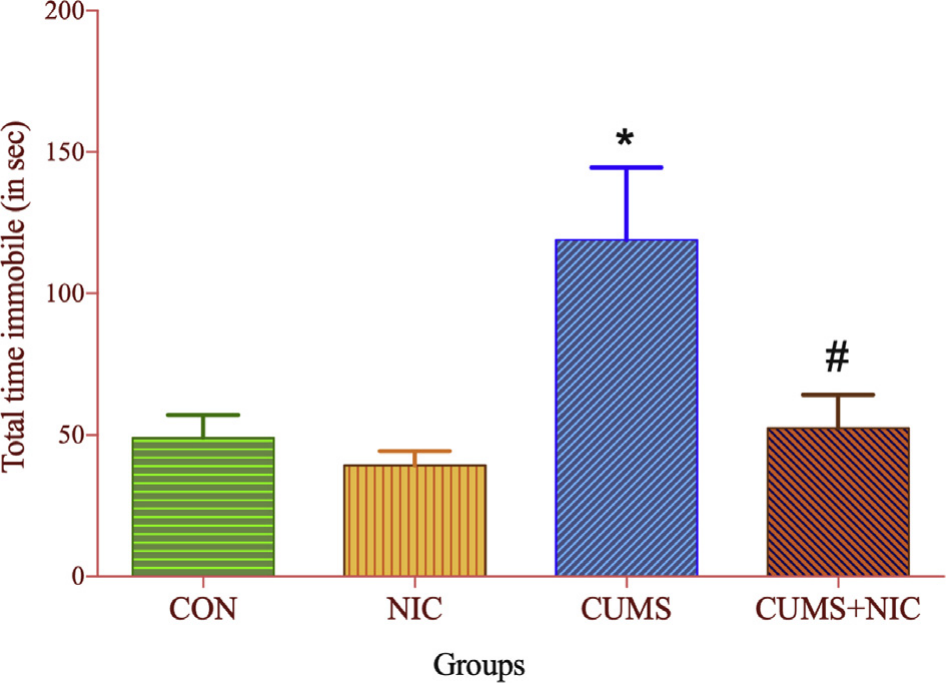
Total time immobile in forced swim test (FST) monitored for 5 min, results are expressed as Mean ± SE (n = 6). Significant difference between CON and CUMS is indicated by *p < 0.05 and by #p < 0.05 between CUMS vs CUMS + NIC group.

FST was used to assess behavioral despair as shown in Fig. 2. In CUMS group total time immobile (Fig. 2) was significantly increased as compared to CON group [F (3, 15) = 6.706, p < 0.05, two-way ANOVA, Tuckey test]. After NIC administration the immobility time was decreased in CUMS + NIC group (p < 0.05).

### 3.2 Nicotine administration increases locomotor activity in CUMS

**Fig. 3:**
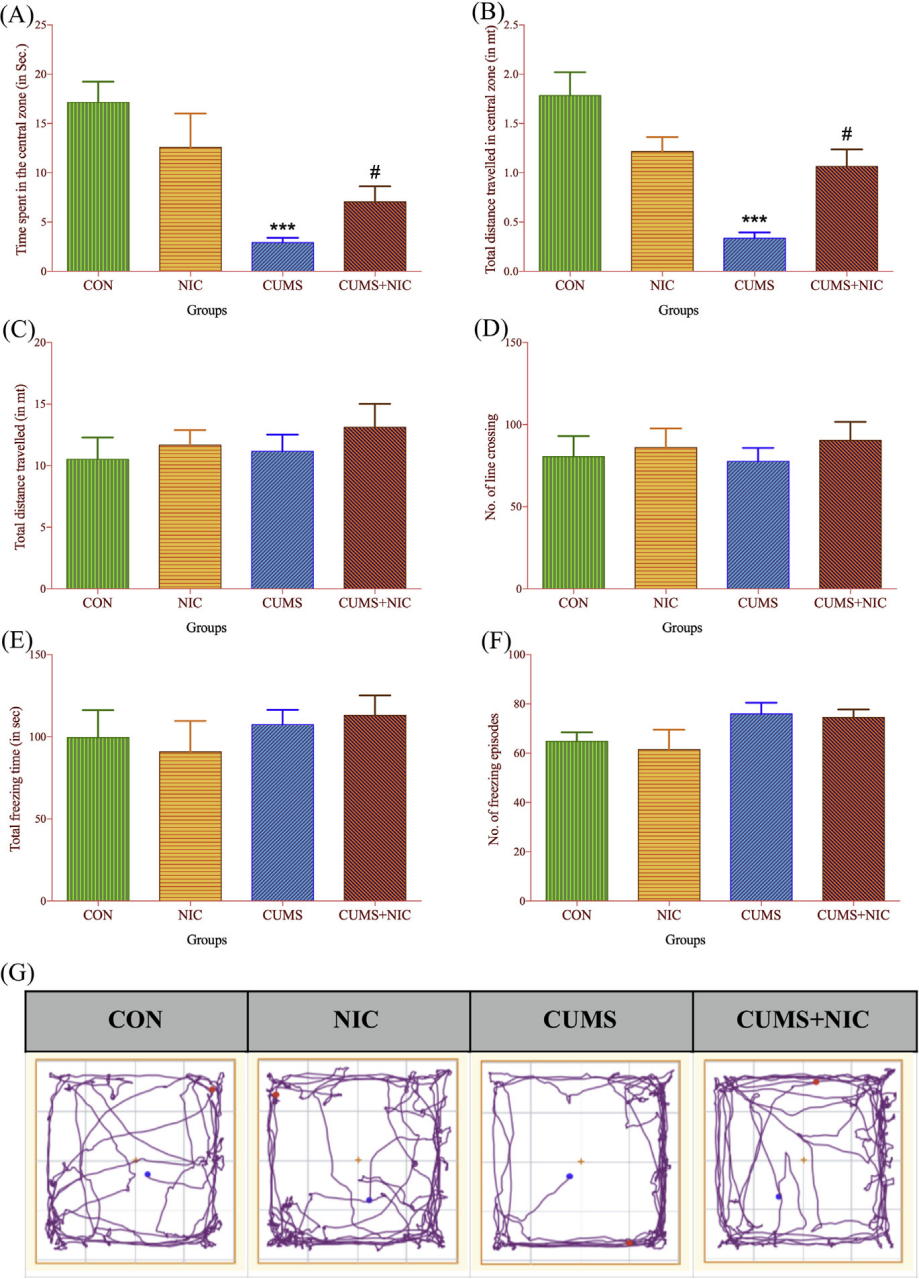
Open field test (OFT) monitored for 5 min. Time spent in the central zone (3A) and total distance travelled in the central zone (3B), results are expressed as Mean ± SE (n = 6). Significant difference between CON and CUMS is indicated by ***p < 0.001 and by #p < 0.05 between CUMS vs CUMS + NIC group. total distance traveled (3C), number of line crossing (3D), total freezing time (3E) and number of freezing episodes (3F) are not significant. (3G) ANY-maze images of open field test.

To assess the effect of CUMS and NIC on locomotor activity OFT was carried out as shown in Fig. 3. Locomotor activity was decreased in the CUMS, which was reversed by NIC administration. Time spent in the central zone (Fig. 3A) was significantly decreased in CUMS group as compared to CON group [F (3, 15) = 8.274, p < 0.001, two-way ANOVA, Tuckey test], and after NIC administration in CUMS + NIC group, it was increased as compared to CUMS group (p < 0.05). Moreover, distance travelled in central zone (Fig. 3B) was significantly less in CUMS group as compared to CON group [F (3, 15) = 14.61, p < 0.001, two-way ANOVA, Tuckey test], which was increased by NIC treatment (p < 0.05). However, we did not get the significance difference in total distance travelled (Fig. 3C) [F (3, 15) = 0.9488], No. of line crossing (Fig. 3D) [F (3, 15) = 0.5121], freezing time (Fig. 3E) [F (3, 15) = 0.7171] and No. of freezing episodes (Fig. 3F) [F (3, 15) = 1.709]. For the statistical analysis, two-way ANOVA and post-hoc Tukey test was used.

### 3.3 Nicotine administration improved memory related impairment associated with CUMS

**Fig. 4:**
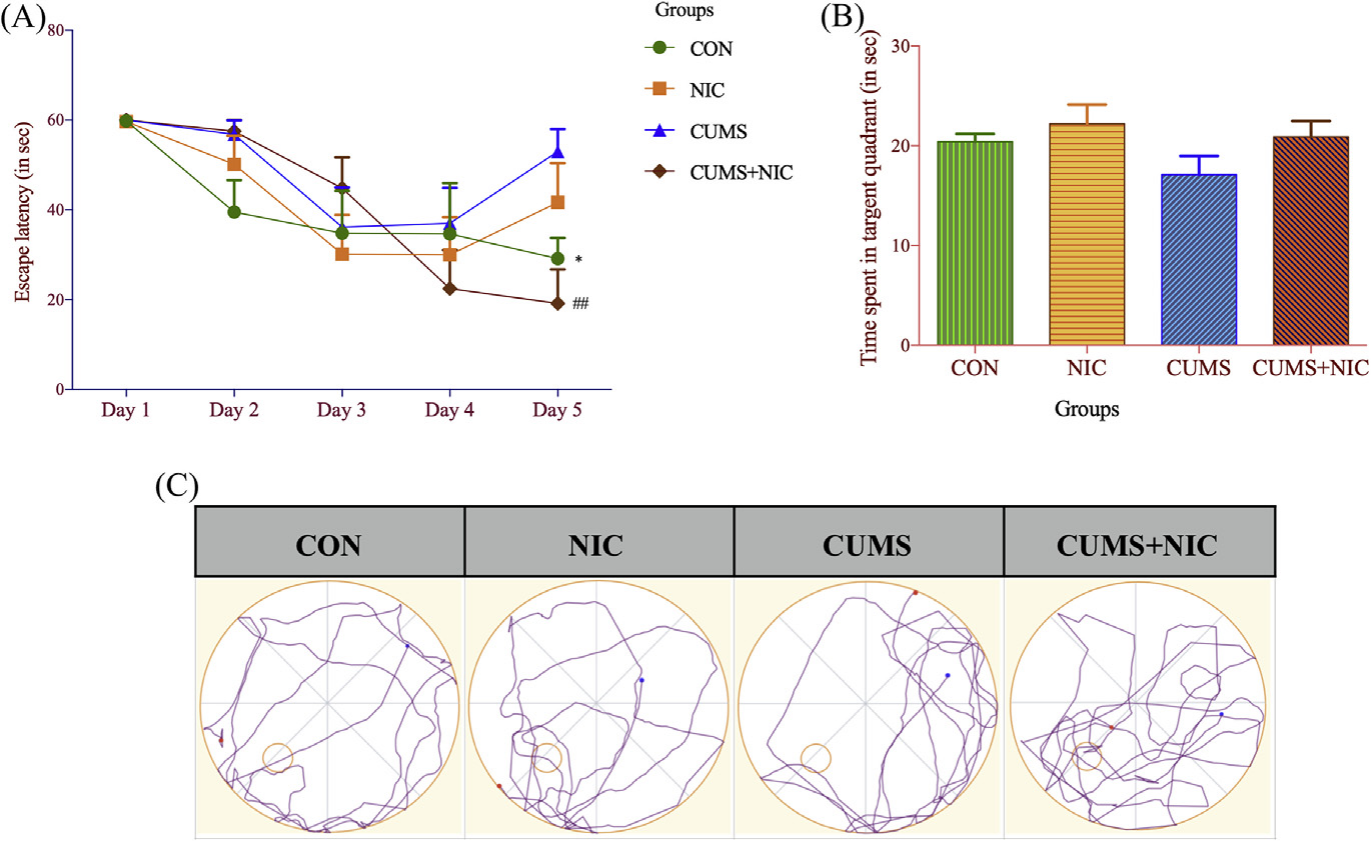
(4A) Escape latency in Morris water maze (MWM), results are expressed as Mean ± SE (n = 6). Significant difference between CON vs CUMS is marked as *p < 0.05 and CUMS vs CUMS + NIC group is indicated by ##p < 0.01, (4B) Time spent in the target quadrant, results are expressed as Mean ± SE (n = 6), Significant difference was not observed. (4C) ANY-maze images of open field test.

To observe the effect of CUMS and NIC on memory and learning we performed Morris water maze (MWM) shown in Fig. 4. In our study we found that CUMS caused altered memory and learning, which was reversed by NIC administration. Two-way ANOVA repeated measures with multiple comparisons revealed a significant increase in the escape latency on the 5^th^ day of trial (Fig. 4A) in CUMS group [F (4, 20) = 8.09, p < 0.05, two-way ANOVA, Tukey test] and treatment with NIC decrease escape latency in CUMS + NIC group (p < 0.01). However, In the final probe trial (Fig. 4B) time spent in the target quadrant was increased in NIC treated group in response to CUMS group but not at the significance level.

### 3.4 Nicotine administration increased BDNF and β-catenin expression in CUMS

**Fig. 5:**
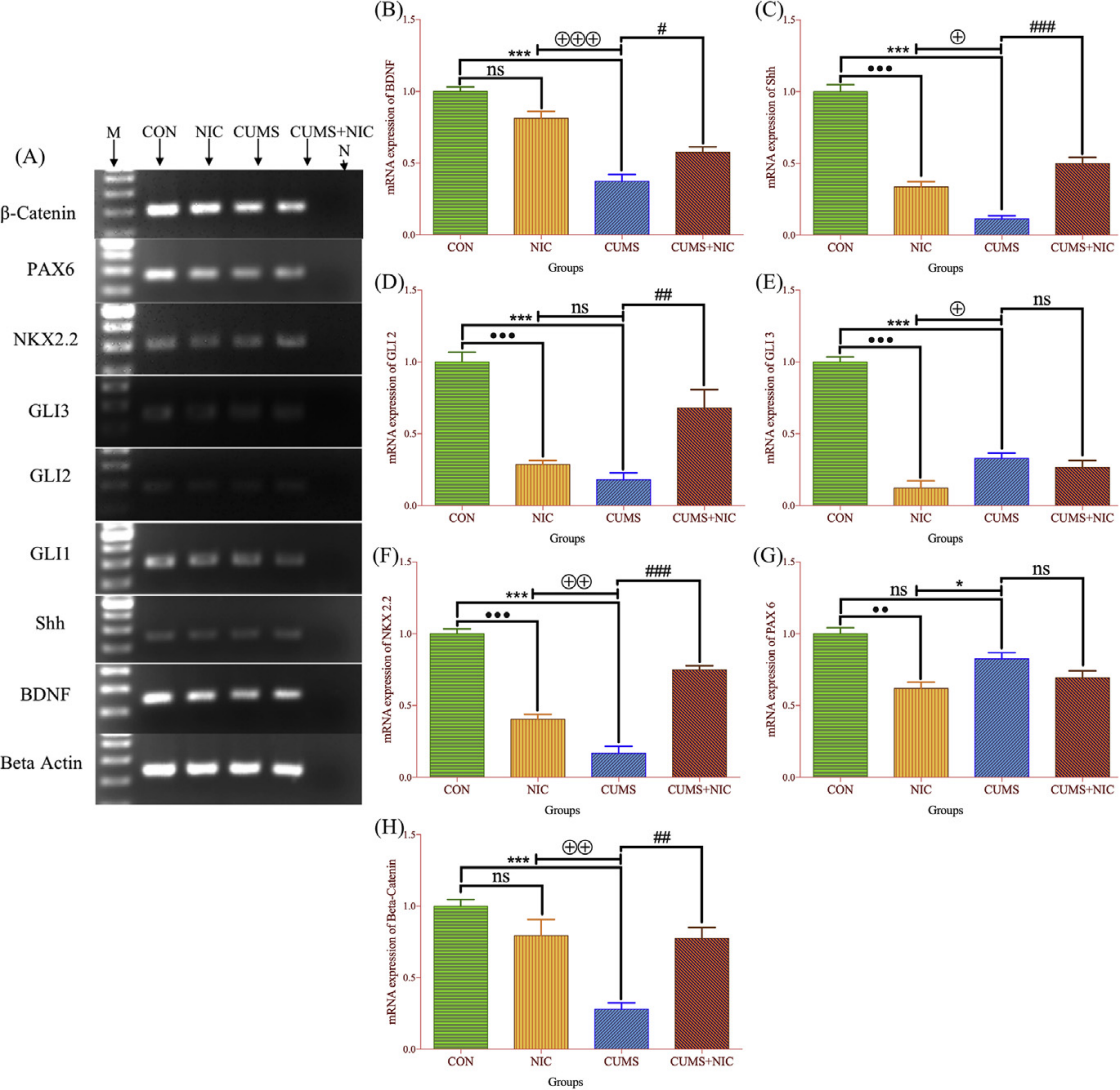
mRNA expression of genes by RT-PCR, β-Actin, BDNF, Shh, GLI1, GLI2, GLI3, NKX2.2, PAX6 and β-catenin in rat hippocampus, all the gels were run separately and grouped as Fig. 5A, M- Marker/DNA ladder of 1000 bp, N-Negative control. The qRT-PCR mRNA expression showed as (5B) BDNF mRNA expression, Shh (5C), GLI2 (5D), GLI3 (5E), NKX2.2 (5F), PAX6 (5G) and β-catenin (5H). Results are expressed as Mean ± SE (n = 3). Significant differences between CON vs NIC is expressed as •; CON vs CUMS is expressed as *; NIC vs CUMS is expressed as ⊕ and CUMS vs CUMS + NIC is expressed by #. p value <0.05 considered as significant.

The RT-PCR (Supplementary Figure S1) showed a decline in expression of BDNF (Supplementary, S1B) and β-catenin (Supplementary, S1F) in CUMS as compared to CON group, however, NIC administration in CUMS increased the expression levels (Fig. 5A). To further examine the mRNA expression of BDNF and β-catenin qRT-PCR results showed CUMS causes decreased expression of BDNF (Fig. 5B) [F (3, 8) = 43.34, p < 0.001, one-way ANOVA, Tukey test] and β-catenin (Fig. 5H) [F (3, 8) = 16.58, p < 0.001, one-way ANOVA, Tukey test] as compared to CON and after NIC administration BDNF mRNA level was restored (p < 0.05) as well as β-catenin expression was also increased (p < 0.01) in the hippocampus of the CUMS exposed rats. BDNF and β-catenin expression was decreased in NIC group but not at the significant level as compared to CON. Moreover, BDNF (p < 0.001) and β-catenin (p < 0.01) expression was significantly higher in NIC group when compared with CUMS group.

### 3.5 Nicotine administration increased Shh signalling cascade in CUMS

RT-PCR (Fig. 5A, Supplementary Figure S1) and qRT-PCR (Figure 5C-G) results showed the effect of CUMS exposure on the hippocampal mRNA expression of Shh (Supplementary, S1C), GLI1 (Supplementary, S1D), GLI2 (Supplementary, S1G), GLI3 (Supplementary, S1E), NKX2.2 (Supplementary, S1H) and PAX6 (Supplementary, S1I). The qRT-PCR results showed that Shh expression (Fig. 5C) significantly downregulates in the CUMS as compared to CON group [F (3, 8) = 94.39, p < 0.001, one-way ANOVA, Tukey test], which was significantly recovered by NIC treatment (p < 0.001). Although, Shh expression also decreased in the NIC group (p < 0.001) but it was significantly higher than the CUMS group (p < 0.05).

We did not notice GLI1 mRNA expression in qRT-PCR. However, in RT-PCR we observed GLI1 expression which is presented in Fig. 5A and in Supplementary Figure S1D. This might be because GLI1 and GLI2 acts as Shh signalling transcriptional activators and showed high similarities with each other.

GLI2 mRNA expression (Fig. 5D) was decreased in CUMS and NIC group [F (3, 8) = 23.7, p < 0.001, one-way ANOVA, Tukey test] as compared to CON, which was significantly increased by NIC treatment (p < 0.01). Although GLI2 was more expressed in NIC than CUMS but not significantly.

GLI3 mRNA expression (Fig. 5E) was decreased in CUMS and NIC group [F (3, 8) = 81.78, p < 0.001, one-way ANOVA, Tukey test] as compared to CON. NIC treatment did not significantly affect GLI3 expression when compared to CUMS. Moreover, GLI3 expression was significantly lower in NIC group than CUMS (p < 0.05).

The expression level of NKX2.2 (Fig. 5F) was significantly decreased in CUMS and NIC groups [F (3, 8) = 98.54, p < 0.001, one-way ANOVA, Tukey test] as compared to CON, which was significantly recovered by NIC treatment (p < 0.001). We also observe significant difference between NIC vs CUMS, in which the expression of NKX2.2 was higher in NIC group than CUMS (p < 0.01).

There was no significant change observed between CON and CUMS in the mRNA expression of PAX6 (Fig. 5G) [F (3, 8) = 14.19, p < 0.09, one-way ANOVA, Tukey test]. Although, PAX6 was significantly decreased in NIC group (p < 0.01) when compared to CON group. Moreover, PAX6 expression was also significantly lower in NIC group than CUMS (p < 0.05).

## 4 Discussion

In this study we attempted to explore the role of Wnt/β-catenin and Shh signalling in the pathophysiology of depression and the involvement of these signalling pathways in providing the antidepressant effect of NIC in male Wistar rats. Moreover, we also observed the ability of NIC to ameliorate cognitive deficits associated with CUMS.

CUMS is the widely accepted animal model of depression [39]. Various studies suggested that CUMS provoke behavioral changes that can be correlated with human depression such as behavioral despair, reduced locomotor activity and decline in learning and memory [40, 41].

First we validated the CUMS rat model of depression by FST [42]. The FST is widely used method to examine depressive behavior and antidepressant effects of the drugs [32, 36, 43]. Depression has been correlated with behavioral despair and measured as immobility time in FST [44]. Various studies showed that acute and chronic NIC administration decreased immobility time in depression models [12, 45]. Moreover, the genetic model of depression namely Flinders Sensitive Line (FSL) rats showed decrease in immobility time after acute and chronic NIC administration [46]. Our study showed that CUMS significantly increased the immobility time and chronic treatment with NIC significantly decreased the immobility time. This is indicating that the depression rat model of CUMS was well established. We also performed OFT, to measure exploratory and anxiety behavior of the rats. The OFT data showed that time spent in central area and distance travelled in the central area was shorter in CUMS group as compared to CON group, which was increased by NIC treatment in CUMS + NIC group. This data suggesting that NIC significantly relieved anxiety symptoms caused by CUMS [43]. However, NIC administration did not affect the total distance travelled, No. of line crossing, total time freezing and No. of freezing episodes in CUMS.

In order to investigate the therapeutic effects of NIC on cognitive deficits caused by CUMS induced depression, MWM was carried out. The data showed increased escape latency caused by CUMS was significantly decreased by NIC treatment on 5^th^ day. Similar to previous studies that suggested NIC enhanced spatial learning and memory [10, 47], we observed an increase in average time spent in target quadrant in the final probe trial, though the data was not significant.

Various evidence supported the hypothesis that BDNF is involved in the etiology of depression [48, 49]. BDNF is a neurotrophin family member which play role in proliferation, embryonic development, differentiation and maturation of neurons [50]. BDNF also involve in adult hippocampal neurogenesis [15]. Clinical and murine studies suggested that BDNF level was decreased in the hippocampus of depressed individual and antidepressant medication restored these BDNF levels [48]. In our study we also quantified the BDNF levels in the hippocampus of male Wistar rats. Our RT-PCR and qRT-PCR data showed decreased mRNA expression of BDNF in CUMS group in relation to CON group. However, this low expression level of BDNF was significantly restored by NIC treatment in CUMS + NIC group. These results were consistent with previous studies [51]. Recently a study reported decreased BDNF expression in the hippocampus due to neurotoxic effect of NIC at high dosage (6 mg/kg/day) [52]. Interestingly, we also observed that NIC given to non-stressed rats also affected the BDNF level but not significantly as we focused on the neuroprotective effects of NIC at low dosage (0.3 mg/kg).

Several reports suggest that BDNF expression is regulated by Wnt signalling in the hippocampus [25]. Furthermore, Wnt signalling is also involved in the neurophysiology of depression [36]. Evidence suggested that Wnt and β-catenin are down regulated in depression [36]. Our results were consistent with the previous studies, which showed that β-catenin expression decreased in depression and NIC administration upregulated β-catenin expression in CUMS, which is the final and central downstream component of Wnt/β-catenin cascade.

There is evidence that suggests decline in hippocampal plasticity and neurogenesis has been associated with the depressive disorder [26]. Shh is a regulator of adult hippocampal neurogenesis [27]. Moreover, a recent study also reported that Shh expression downregulates in the CUMS induced prenatal stress in the hippocampus [24]. We also observe downregulation of Shh in CUMS induced depression. Interestingly, NIC also affect the Shh pathway and NIC treatment upregulate the depleted Shh level. Moreover, we also observed that NIC alone also causing negative effect on the Shh pathway, Although Shh expression in the NIC group was higher than CUMS group. As this is the first study showed the effect of NIC on the Shh pathway. However, further research is required to understand more about this basic developmental pathway and its regulation by NIC in the brain.

We also observed mRNA expression of Shh signalling components or downstream transcription factors, namely, GLI1/2/3, NKX2.2 and PAX6. The primary downstream Shh signalling components are GLI1/2, which are released from the smoothened receptor after binding of Shh ligand to Patch1 receptor [28, 53]. Further, this GLI1/2 enters into the nucleus and binds to transcription factors including N-myc, Cyclin D2, Plakoglobin, NKX2.2 and PAX6 to regulates their expression [53]. Moreover, GLI1/2 are considered as transcriptional activators and GLI3 is regarded as a transcriptional repressor of Shh cell signalling pathway [53, 54, 55]. A recent study reported that GLI1 and GLI2 transcriptional activity overlap each other in neoplastic chondrocytes [56]. Intrestingly, GLI2 transcription factor was more active in the absence of GLI1 and activate Shh pathway [57]. Although, we did not observe GLI1 expression but the pathway might be activated by GLI2 in the hippocampus of male Wistar rats.

NKX2.2 and PAX6 are the homeodomain transcription factors of Shh signalling pathway. Various studies showed the role of these two transcription factors in the embryonic neural development and in the brain tumor growth [28, 53, 58]. We observed declined NKX2.2 expression in CUMS and treatment with NIC restored this decreased NKX2.2 expression level. Although this expression was also decreased in NIC group but it was higher in NIC than CUMS group which means CUMS affecting the pathway more than NIC. The reason may be, in providing antidepressant effect the signalling become more active as a healing or recovery process. We did not observe any significant difference in PAX6 expression in CUMS. Although, NIC decreased the PAX6 expression level. There are studies which explains the graded role of NKX2.2 and PAX6 in Shh signalling [58].

Shh is transported to hippocampus anterogradely though GABAergic neurons, however, NIC provides its rewarding and motivational effects through dopaminergic and GABAergic neurons in VTA. GABAergic neurons also regulate hippocampal plasticity, so there might be the possibility that Shh also plays role in the reward mechanism in NIC action [59]. Nevertheless, it is further needed to understand the molecular mechanism of Shh cell signalling pathway role in depression and in NIC addiction. In the current study, we are reporting a novel role of Shh cell signalling in CUMS rat model and also its involvement in the NIC facilitated antidepressant effect. Although the current study has been performed in animals, the model used here is widely accepted as an ideal system to study antidepressant effect of various potential therapeutic agents. However, the effect of nicotine on Shh signalling in humans needs to be further elucidated.

## 5 Conclusion

To conclude our study, we suggest that BDNF, Wnt/β-catenin and Shh signalling plays important role in the pathophysiology of CUMS induced depression and in providing the NIC mediated antidepressant effect.

## Declarations

### Author contribution statement

Mohd Tayyab: Conceived and designed the experiments; Performed the experiments; Analyzed and interpreted the data; Wrote the paper.

M. Mobarak Hossain, Mehdi H. Shahi: Conceived and designed the experiments; Contributed reagents, materials, analysis tools or data; Wrote the paper.

Shirin Farheen, Mubeena Mariyath P.M, Nabeela Khanam: Analyzed and interpreted the data.

### Funding statement

This work was supported by Interdisciplinary Brain Research Centre (IBRC), J.N. Medical College, Faculty of Medicine, Aligarh Muslim University, Aligarh, Uttar Pradesh 202002, India.

### Competing interest statement

The authors declare no conflict of interest.

### Additional information

No additional information is available for this paper.

## References

[1] Zhang M., Xu W., He G., Zhang D., Zhao X., Dai J., Wu J., Cao Y., Wang Z., Wang L., Qiao Z. (2018). Maternal nicotine exposure has severe cross-generational effects on offspring behavior. Behav. Brain Res..

[2] WHO report on the global tobacco epidemic (2017). Monitoring Tobacco Use and Prevention Policies.

[3] Mathew A.R., Hogarth L., Leventhal A.M., Cook J.W., Hitsman B. (2017). Cigarette smoking and depression comorbidity: systematic review and proposed theoretical model. Addiction.

[4] W.H. Organization (2017). Depression and other common mental disorders: global health estimates.

[5] Perkins K.A., Grobe J.E. (1992). Increased desire to smoke during acute stress. Br. J. Addict..

[6] Laviolette S.R., van der Kooy D. (2004). The neurobiology of nicotine addiction: bridging the gap from molecules to behaviour,. Nat. Rev. Neurosci..

[7] Iniguez S.D., Warren B.L., Parise E.M., Alcantara L.F., Schuh B., Maffeo M.L., Manojlovic Z., Bolanos-Guzman C.A. (2009). Nicotine exposure during adolescence induces a depression-like state in adulthood. Neuropsychopharmacology.

[8] Wang J., Jia Y., Li G., Wang B., Zhou T., Zhu L., Chen T., Chen Y. (2018). The dopamine receptor D3 regulates lipopolysaccharide-induced depressive-like behavior in mice,. Int. J. Neuropsychopharmacol..

[9] Berrendero F., Plaza-Zabala A., Galeote L., Flores A., Bura S.A., Kieffer B.L., Maldonado R. (2012). Influence of delta-opioid receptors in the behavioral effects of nicotine. Neuropsychopharmacology.

[10] Gandelman J.A., Newhouse P., Taylor W.D. (2018). Nicotine and networks: potential for enhancement of mood and cognition in late-life depression. Neurosci. Biobehav. Rev..

[11] Knott V., Thompson A., Shah D., Ilivitsky V. (2012). Neural expression of nicotine's antidepressant properties during tryptophan depletion: an EEG study in healthy volunteers at risk for depression. Biol. Psychol..

[12] Tizabi Y., Overstreet D.H., Rezvani A.H., Louis V.A., Clark E., Janowsky D.S., Kling M.A. (1999). Antidepressant effects of nicotine in an animal model of depression. Psychopharmacology (Berl).

[13] Pekala K., Michalak A., Kruk-Slomka M., Budzynska B., Biala G. (2018). Impacts of cannabinoid receptor ligands on nicotine- and chronic mild stress-induced cognitive and depression-like effects in mice. Behav. Brain Res..

[14] Alkadhi K.A. (2018). Neuroprotective effects of nicotine on hippocampal long-term potentiation in brain disorders. J. Pharmacol. Exp. Ther..

[15] Lee E., Son H. (2009). Adult hippocampal neurogenesis and related neurotrophic factors. BMB Rep..

[16] Castren E., Rantamaki T. (2010). Role of brain-derived neurotrophic factor in the aetiology of depression: implications for pharmacological treatment. CNS Drugs.

[17] Bjorkholm C., Monteggia L.M. (2016). BDNF - a key transducer of antidepressant effects. Neuropharmacology.

[18] Voleti B., Duman R.S. (2012). The roles of neurotrophic factor and Wnt signaling in depression,. Clin. Pharmacol. Ther..

[19] Madsen T.M., Newton S.S., Eaton M.E., Russell D.S., Duman R.S. (2003). Chronic electroconvulsive seizure up-regulates beta-catenin expression in rat hippocampus: role in adult neurogenesis. Biol. Psychiatry.

[20] Teo C.H., Soga T., Parhar I.S. (2018). Brain Beta-Catenin Signalling During Stress and Depression. Neurosignals.

[21] Patel S.S., Mahindroo N., Udayabanu M. (2016). Urtica dioica leaves modulates hippocampal smoothened-glioma associated oncogene-1 pathway and cognitive dysfunction in chronically stressed mice. Biomed. Pharmacother..

[22] Rajendran R., Jha S., Fernandes K.A., Banerjee S.B., Mohammad F., Dias B.G., Vaidya V.A. (2009). Monoaminergic regulation of Sonic hedgehog signaling cascade expression in the adult rat hippocampus. Neurosci. Lett..

[23] Banerjee S.B., Rajendran R., Dias B.G., Ladiwala U., Tole S., Vaidya V.A. (2005). Recruitment of the Sonic hedgehog signalling cascade in electroconvulsive seizure-mediated regulation of adult rat hippocampal neurogenesis. Eur. J. Neurosci..

[24] Fatima M., Srivastav S., Ahmad M.H., Mondal A.C. (2019). Effects of chronic unpredictable mild stress induced prenatal stress on neurodevelopment of neonates: role of GSK-3beta. Sci. Rep..

[25] Yi H., Hu J., Qian J., Hackam A.S. (2012). Expression of brain-derived neurotrophic factor is regulated by the Wnt signaling pathway. Neuroreport.

[26] Tayyab M., Shahi M.H., Farheen S., Mariyath M.P.M., Khanam N., Castresana J.S., Hossain M.M. (2018). Sonic hedgehog, Wnt, and brain-derived neurotrophic factor cell signaling pathway crosstalk: potential therapy for depression. J. Neurosci. Res..

[27] Yao P.J., Petralia R.S., Mattson M.P. (2016). Sonic Hedgehog Signaling and Hippocampal Neuroplasticity. Trends Neurosci..

[28] Shahi M.H., Rey J.A., Castresana J.S. (2012). The sonic hedgehog-GLI1 signaling pathway in brain tumor development,. Expert Opin. Ther. Targets.

[29] Kalejaiye O., Bhatti B.H., Taylor R.E., Tizabi Y. (2013). Nicotine blocks the depressogenic effects of alcohol: implications for drinking-smoking Co-morbidity. J. Drug Alcohol Res..

[30] Biala G., Pekala K., Boguszewska-Czubara A., Michalak A., Kruk-Slomka M., Budzynska B. (2017). Behavioral and biochemical interaction between nicotine and chronic unpredictable mild stress in mice. Mol. Neurobiol..

[31] Dubey V.K., Ansari F., Vohora D., Khanam R. (2015). Possible involvement of corticosterone and serotonin in antidepressant and antianxiety effects of chromium picolinate in chronic unpredictable mild stress induced depression and anxiety in rats. J. Trace Elem. Med. Biol..

[32] Porsolt R.D., Le Pichon M., Jalfre M. (1977). Depression: a new animal model sensitive to antidepressant treatments. Nature.

[33] Firdaus F., Zafeer M.F., Ahmad M., Afzal M. (2018). Anxiolytic and anti-inflammatory role of thymoquinone in arsenic-induced hippocampal toxicity in Wistar rats. Heliyon.

[34] Romero R.D., Chen W.J. (2004). Gender-related response in open-field activity following developmental nicotine exposure in rats. Pharmacol. Biochem. Behav..

[35] Liu D., Wang Z., Gao Z., Xie K., Zhang Q., Jiang H., Pang Q. (2014). Effects of curcumin on learning and memory deficits, BDNF, and ERK protein expression in rats exposed to chronic unpredictable stress. Behav. Brain Res..

[36] Yang X.H., Song S.Q., Xu Y. (2017). Resveratrol ameliorates chronic unpredictable mild stress-induced depression-like behavior: involvement of the HPA axis, inflammatory markers, BDNF, and Wnt/beta-catenin pathway in rats. Neuropsychiatric Dis. Treat..

[37] Shen J., Xu L., Qu C., Sun H., Zhang J. (2018). Resveratrol prevents cognitive deficits induced by chronic unpredictable mild stress: Sirt1/miR-134 signalling pathway regulates CREB/BDNF expression in hippocampus in ​vivo and in ​vitro. Behav. Brain Res..

[38] Schmittgen T.D., Livak K.J. (2008). Analyzing real-time PCR data by the comparative C(T) method. Nat. Protoc..

[39] Willner P., Muscat R., Papp M. (1992). Chronic mild stress-induced anhedonia: a realistic animal model of depression. Neurosci. Biobehav. Rev..

[40] Gronli J., Murison R., Fiske E., Bjorvatn B., Sorensen E., Portas C.M., Ursin R. (2005). Effects of chronic mild stress on sexual behavior, locomotor activity and consumption of sucrose and saccharine solutions. Physiol. Behav..

[41] Song L., Che W., Min-Wei W., Murakami Y., Matsumoto K. (2006). Impairment of the spatial learning and memory induced by learned helplessness and chronic mild stress. Pharmacol. Biochem. Behav..

[42] Biala G., Pekala K., Boguszewska-Czubara A., Michalak A., Kruk-Slomka M., Grot K., Budzynska B. (2018). Behavioral and Biochemical Impact Of chronic unpredictable mild stress on the acquisition of nicotine conditioned place preference in rats. Mol. Neurobiol..

[43] Xiao X., Shang X., Zhai B., Zhang H., Zhang T. (2018). Nicotine alleviates chronic stress-induced anxiety and depressive-like behavior and hippocampal neuropathology via regulating autophagy signaling. Neurochem. Int..

[44] Lino-de-Oliveira C., De Lima T.C., de Padua Carobrez A. (2005). Structure of the rat behaviour in the forced swimming test. Behav. Brain Res..

[45] Suemaru K., Yasuda K., Cui R., Li B., Umeda K., Amano M., Mitsuhashi H., Takeuchi N., Inoue T., Gomita Y., Araki H. (2006). Antidepressant-like action of nicotine in forced swimming test and brain serotonin in mice. Physiol. Behav..

[46] Tizabi Y., Rezvani A.H., Russell L.T., Tyler K.Y., Overstreet D.H. (2000). Depressive characteristics of FSL rats: involvement of central nicotinic receptors. Pharmacol. Biochem. Behav..

[47] Shang X., Shang Y., Fu J., Zhang T. (2017). Nicotine significantly improves chronic stress-induced impairments of cognition and synaptic plasticity in mice. Mol. Neurobiol..

[48] Bai Y., Song L., Dai G., Xu M., Zhu L., Zhang W., Jing W., Ju W. (2018). Antidepressant effects of magnolol in a mouse model of depression induced by chronic corticosterone injection. Steroids.

[49] Motamedi S., Karimi I., Jafari F. (2017). The interrelationship of metabolic syndrome and neurodegenerative diseases with focus on brain-derived neurotrophic factor (BDNF): kill two birds with one stone,. Metab. Brain Dis..

[50] Henderson C.E. (1996). Role of neurotrophic factors in neuronal development. Curr. Opin. Neurobiol..

[51] Machaalani R., Chen H. (2018). Brain derived neurotrophic factor (BDNF), its tyrosine kinase receptor B (TrkB) and nicotine. Neurotoxicology.

[52] Motaghinejad M., Motevalian M., Fatima S., Faraji F., Mozaffari S. (2017). The neuroprotective effect of curcumin against nicotine-induced neurotoxicity is mediated by CREB-BDNF signaling pathway,. Neurochem. Res..

[53] Shahi M.H., Afzal M., Sinha S., Eberhart C.G., Rey J.A., Fan X., Castresana J.S. (2010). Regulation of sonic hedgehog-GLI1 downstream target genes PTCH1, Cyclin D2, Plakoglobin, PAX6 and NKX2.2 and their epigenetic status in medulloblastoma and astrocytoma. BMC Canc..

[54] Altaba A. Ruiz i (1999). Gli proteins encode context-dependent positive and negative functions: implications for development and disease. Development.

[55] Altaba A. Ruiz i, Palma V., Dahmane N. (2002). Hedgehog-Gli signalling and the growth of the brain. Nat. Rev. Neurosci..

[56] Ali S.A., Niu B., Cheah K.S.E., Alman B. (2019). Unique and overlapping GLI1 and GLI2 transcriptional targets in neoplastic chondrocytes. PLoS One.

[57] Bai C.B., Auerbach W., Lee J.S., Stephen D., Joyner A.L. (2002). Gli2, but not Gli1, is required for initial Shh signaling and ectopic activation of the Shh pathway. Development.

[58] Balaskas N., Ribeiro A., Panovska J., Dessaud E., Sasai N., Page K.M., Briscoe J., Ribes V. (2012). Gene regulatory logic for reading the Sonic Hedgehog signaling gradient in the vertebrate neural tube. Cell.

[59] Luo S.X., Huang E.J. (2016). Dopaminergic Neurons and Brain Reward Pathways: from neurogenesis to circuit assembly. Am. J. Pathol..

