# Supplementary material for: Exploring the potential role of sonic hedgehog cell signalling pathway in antidepressant effects of nicotine in chronic unpredictable mild stress rat model
Source: Heliyon. 2019 May 10;5(5):e01600. doi: 10.1016/j.heliyon.2019.e01600 (PMC6514495; doi:10.1016/j.heliyon.2019.e01600)
Supplement: Supplementary material [file mmc1.docx]

**Supplementary Material**


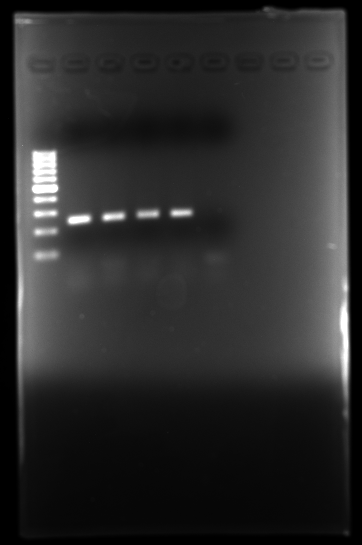

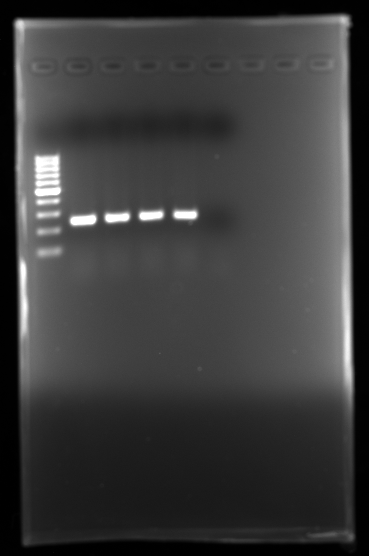


**(S1A)**

CON

L

NIC

CUMS

CUMS+NIC

N

CON

L

NIC

CUMS

CUMS+NIC

N

**(S1B)**

BDNF

Beta-Actin


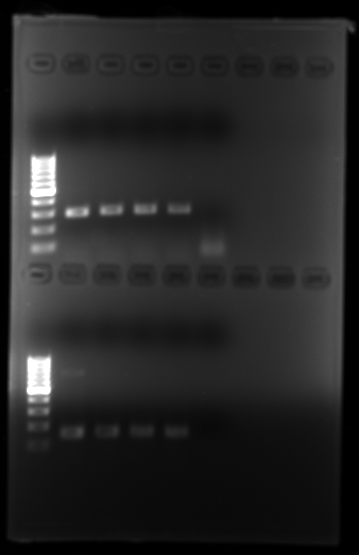

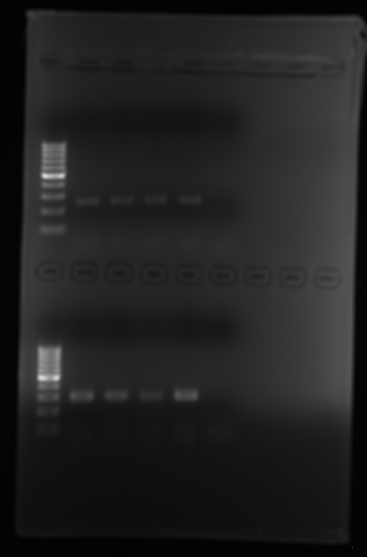


GLI3

**(S1E)**

GLI1

CON

L

NIC

CUMS

CUMS+NIC

N

CON

L

NIC

CUMS

CUMS+NIC

N

**(S1C)**

**(S1D)**

Shh

NKX2.2 Not included in the manuscript

CON

L

NIC

CUMS

CUMS+NIC

N

CON

L

NIC

CUMS

CUMS+NIC

N

**(S1H)**


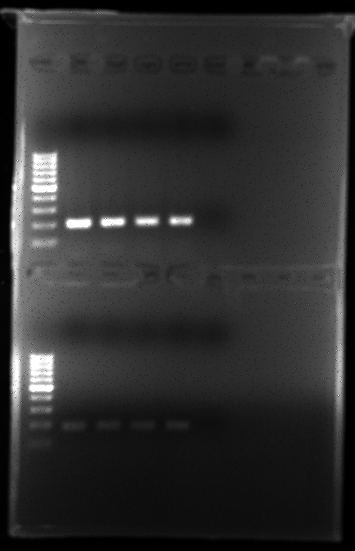

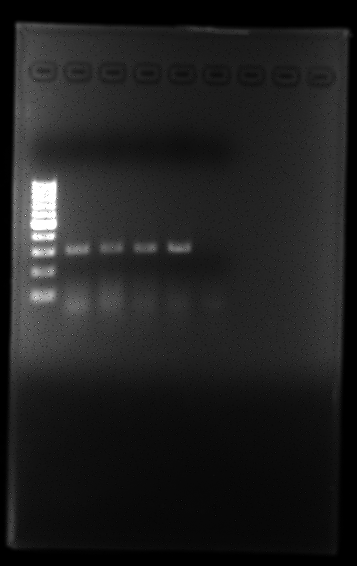


**(S1F)**

Beta-Catenin

NKX2.2

**(S1G)**

GLI2


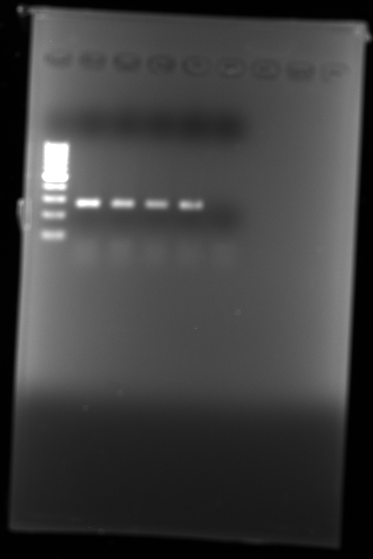


**(S1I)**

PAX6

CON

L

NIC

CUMS

CUMS+NIC

N

Figure S1

**Figure S1**. Gels of mRNA expression by RT-PCR, β-Actin (S1A), BDNF (S1B), Shh (S1C), GLI1 (S1D), GLI2 (S1G), GLI3 (S1E), NKX2.2 (S1H), PAX6 (S1I) and β-catenin (S1F) in rat hippocampus, M- Marker/DNA ladder of 1000 bp, N-Negative control.

**Highlights:**

1. Nicotine (NIC) may provide relief effect from the symptoms of depression and anxiety, which might be the cause of its addiction in adults.
2. NIC administration also results in cognitive enhancement.
3. The low BDNF expression in the hippocampus due to depression elevated by NIC administration.
4. NIC also showed positive effect on the expression of Shh, GLI1/2/3, NKX2.2 and β-catenin.
5. Our study suggests the involvement of Shh and Wnt/β-catenin signalling in depression.
